# First report on metabarcoding analysis of gut microbiome in Island Flying Fox (*Pteropushypomelanus*) in island populations of Malaysia

**DOI:** 10.3897/BDJ.10.e69631

**Published:** 2022-03-22

**Authors:** Nur Syafika Mohd-Yusof, Muhammad Abu Bakar Abdul-Latiff, Abd Rahman Mohd-Ridwan, Aqilah Sakinah Badrulisham, Nursyuhada Othman, Salmah Yaakop, Shukor Md-Nor, Badrul Munir Md-Zain

**Affiliations:** 1 Department of Biological Sciences and Biotechnology, Faculty of Science and Technology, Universiti Kebangsaan Malaysia, 43600, Bangi, Selangor, Malaysia Department of Biological Sciences and Biotechnology, Faculty of Science and Technology, Universiti Kebangsaan Malaysia, 43600 Bangi, Selangor Malaysia; 2 Faculty of Applied Sciences and Technology Universiti Tun Hussein Onn Malaysia (Pagoh Campus), KM1 Jalan Panchor 84600, Muar, Johor, Malaysia Faculty of Applied Sciences and Technology Universiti Tun Hussein Onn Malaysia (Pagoh Campus), KM1 Jalan Panchor 84600 Muar, Johor Malaysia; 3 Centre for Pre-University Studies, Universiti Malaysia Sarawak, 94300, Kota Samarahan, Sarawak, Malaysia Centre for Pre-University Studies, Universiti Malaysia Sarawak, 94300 Kota Samarahan, Sarawak Malaysia

**Keywords:** Flying Fox, *Pteropushypomelanus*, gut microbiome, high-throughput sequencing, metabarcoding

## Abstract

Flying fox (*Pteropushypomelanus*) belongs to the frugivorous bats, which play a crucial role in maintaining proper functioning of an ecosystem and conservation of the environment. Bats are well-known carriers of pathogenic viruses, such as BatCov RaTG13 from the coronavirus family that share 90.55% with SARS-CoV-2, the pathogen causing recent global pandemic coronavirus disease 19 (COVID-19). However, bats’ possible role as a carrier of pathogenic bacteria is less explored. Here, using metabarcoding analysis through high-throughput sequencing, we explored the gut microbiome composition of different island populations on the east and west coasts of Peninsula Malaysia. The *16S* rRNA gene in samples from Redang Island, Langkawi Island, Pangkor Island and Tinggi Island was amplified. Bacterial community composition and structure were analysed with α and β diversity metrics. A total of 25,658 operational taxonomic units at 97% similarity were assigned to eight phyla, 44 families, 61 genera and 94 species of microbes. The Proteobacteria was the dominant phylum in all populations. Meanwhile, the genera *Enterobacter*, *Pseudomonas* and *Klebsiella*, isolated in this study, were previously found in the rectum of other fruit bats. Our analyses suggest that Redang Island and Langkawi Island have high bacteria diversity. Thus, we found geographic locality is a strong predictor of microbial community composition and observed a positive correlation between ecological features and bacterial richness.

## Introduction

Bats are capable of flying high and covering long distances during seasonal migrations. Bats have extraordinary adaptation and are able to inhabit a huge number of diverse ecological niches (Mühldorfer (2013). These characteristics have led to increased global interest in bats as potential reservoir hosts and vectors of zoonotic pathogens (Kuzmin et al. 2011, Wang et al. 2011). The current global pandemic indicates that bats and pangolins are amongst wildlife associated with the spread of human coronavirus that causes COVID-19 (Cui et al. 2019, Zhang et al. 2020). In addition, frugivorous bats play a crucial role in dispersing seedlings and maintaining forest tree diversity and regeneration (Muscarella and Fleming 2007). A recent study by Aziz et al. (2017) reported that one of the most important crops in Southeast Asia, durian (*Duriozibethinus*), depends largely on the Island Flying Fox during pollination. By examining the gut microbiota of Island Flying Fox, the findings are predicted to give better understanding on how the microbial communities amongst Island Flying Fox can serve as a basis for assessing its ecology, evolution and health status.

Early microbiome studies on wildlife focused on primates which represent only 5% of the diversity of extant mammals (Wilson and Reeder 2005). The gut microbiome refers to the bacteria found in the intestinal tract. Studies on the human gut microbiome have found that the gut microbiome has a larger influence on various aspects of host health than initially thought. Every individual has a unique gut microbiota profile that plays a role in host nutrient metabolism, immunity, and protection against pathogens. It acts as a barrier against harmful microbes by competing for nutrients and ecological binding sites (Buffie and Pamer 2013) and producing antimicrobial substances (Ubeda et al. 2013). Recent technological advances have led to an increasing number of gut microbiome studies. However, many studies in the field investigate the role of gut microbiome in human, domestic animals or laboratory models. Therefore, information on microbiome functions in mammalians is limited to only a few taxa, leaving the broader patterns of microbiome function and evolution unexplored (Ingala et al. 2018).

Previous studies found that Henipaviruses were the most common zoonotic pathogens in natural reservoir hosts, species of *Pteropus* (Mackenzie et al. 2003, Daszak et al. 2006). Bacterial communities, associated with dietary habits of bats, were less explored (Phillips et al. 2012). Their role of microbiome in disease epidemiology is important as bats are prone to a variety of microorganisms including viruses, bacteria, fungi and parasites (Whitaker Jr et al. 2009, Wibbelt et al. 2009). However, microbiota composition in the known 1,411 species of bats (Wilson and Mittermeier 2019), has not been well studied and, thus, should be explored in order to understand bats’ microbiome from various aspects including host-microbe relationships (Daniel et al. 2013, Banskar et al. 2016b). Similarly, Banskar et al. (2016b) reported that bats carry potential bacterial pathogens and suggested the importance of studying the effect of these pathogens on bats themselves and possible transmission to humans and other animals.

Fruits are the primary diet of bats and influence the abundance of different types of bacteria in their intestinal tract due to the nutritionally-rich environment, whose bacterial composition provides information about the dietary habits and feeding behaviour of bats (David et al. 2014, Banskar et al. 2016a). Foraging habits may be critical for richness of gut bacteria and such information is necessary in determining the ecological significance of the hosts (Mühldorfer 2013, Daniel et al. 2013). In addition, a report by Sharon et al. (2010) suggested that gut microbes not only affect host physiology, but also affect their evolution.

Conventional methods, such as microbial cultivation is considered inefficient, time consuming and inadequate (Hilton et al. 2016). Only 10 to 30 percent of gut microbiota were successfully cultivated using this method, which fails to recover the majority of the microbial species in the host animal (Tannock 2001). The revolution of 16S rRNA sequencing has become an effective approach to reveal the uncultivated gut microbial communities (Wang et al. 2015). The application of 16S rRNA in molecular technique has been widely used to characterise gut microbial communities in wildlife such as bats, primates and tigers (Phillips et al. 2012, Carrillo-Araujo et al. 2015, Chen et al. 2018). Further, the 16S gene comprises nine hypervariable (V1-V9) regions and sequence dissimilarity amongst bacteria within these regions allows researchers to identify and differentiate organisms taxonomically (Pace 1997). It has been shown that two variant regions in 16S rRNA gene, namely V3 and V4, provide sufficient phylogenetic information on bacteria (Liu et al. 2007, Huse et al. 2008). For example, by using the culture-based approach, researchers have used this gene to identify bacterial species in the intestine of the bat *Cynopterusbrachyotis*, providing more detailed information than previous studies at only the genus level (Daniel et al. 2013).

As information on the distribution and diversity of microbes found in pteropodids from the Indomalaya Region is limited (Daniel et al. 2013), the present study focuses on identification and screening of the bacterial communities from bats’ intestine samples using next-generation sequencing (NGS). The results provide an overview of bacteria isolated from bats with a particular emphasis on major bacterial pathogens that have the potential to cause disease in bats and humans. In addition, dietary habits and geographical locations of the Island Flying Fox have been discussed as factors affecting their potential as vectors of pathogenic bacteria.

## Material and methods

### Study area

**Figure 1.**
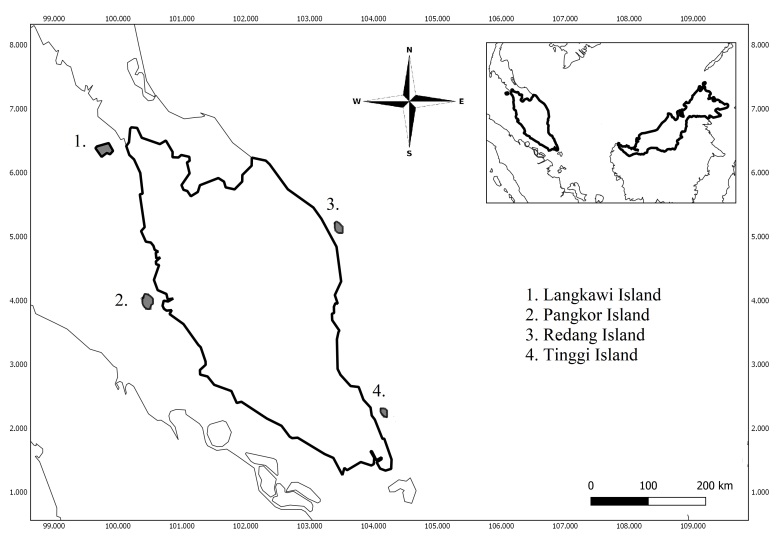
Map of sampling locations of *P.hypomelanus* in Peninsular Malaysia.

In Peninsular Malaysia, we sampled *P.hypomelanus* at four sites, including Dangli Island (Langkawi), Pantai Teluk Nipah in Pangkor Island (Perak), Mat Kepit in Redang Island (Terengganu) and Tanjung Balang in Tinggi Island (Johor) (Fig. 1).

Dangli Island

Dangli Island is a small island to the north of Langkawi Island. The uninhabited Island is visible from the beach at Tanjung Rhu. It is within a small group of islets that include Gasing Island and Pasir Island. *Pteropushypomelanus* can be found roosting in the trees on the two rocky outcrops on Dangli Island in the north and Lalang Island to the south of Langkawi Archipelago. This Island is classified as the most environmentally diverse amongst all sites. Its inland is mountainous and covered with mixed dipterocarp forest.

Pangkor Island

Pangkor Island is an island located in the Strait of Malacca on the west coast of Peninsular Malaysia between 04°13.0’N latitude and 100°33.0’E longitude. It is one of the most famous islands in Malaysia, 3.5 km from Peninsular Malaysia with a land area of 18 km^2^. This Island is classified as coastal hill forest with high conservation. The highest point is Bukit Pangkor at 371 m a.s.l. It has a population of approximately 25,000 and the major industries of the Island are tourism and fishing.

Redang Island

Redang Island is an island in Kuala Nerus District, Terengganu, Malaysia. It is one of the largest islands off the east coast of Peninsular Malaysia, which lies about 45 km from the coast of Terengganu State. Pulau Redang measures about 7 km long and 6 km wide. Its highest peak is Bukit Besar at 359 m a.s.l. It has an equatorial climate with high temperatures throughout the year, ranging from 22°C in the early morning to 34°C at noon. This Island is an important conservation site for sea turtles.

Tinggi Island

Tinggi Island is located 37 km southeast of Mersing, on the east coast of Johor with a land area of 15 km^2^. It rises 600 m a.s.l. It takes approximately 45 minutes to reach Tinggi Island by boat from the mainland. The inland is mostly covered by secondary lowland Dipterocarp rainforest. It has fresh water, fruits, rattan and timber. A sheltered harbour with coral reefs abounds with prolific marine life. It has a long coastline with white sandy beaches and caves. This Island has the highest residential population amongst the east coast Johor Islands, with the latest tally estimated at only 448 people, from three villages including Kampung Tanjung Balang, Kampung Pasir Panjang and Kampung Sebirah Besar.

### DNA extraction and 16S rRNA gene amplification

Bacterial genomic DNA extraction was carried out using innuPREP Stool DNA Kit (Analytik Jena, Germany). The purity and concentration of DNA were confirmed by both spectrophotometric and fluorometric methods using Implen Nanophotometer and Qubit 4.0 HS Assay Kit (Life Technologies), respectively. Two rounds of polymerase chain reaction (PCR) were performed. The first PCR is amplification of the targeted locus of 16S rRNA gene. The second PCR is for indexing of purified PCR products. 16S rRNA gene was amplified by PCR using primers targeting the V3 and V4 regions. The following are the universal bacterial primer pair F515 (5’-TCGTCGGCAGCGTCAGATGTGTATAAGAGACAGGTGCCAGCMGCCGCGGTAA-3’) and R806 (5’-GTCTCGTGGGCTCGGAGATGTGTATAAGAGACAGGGACTACHVGGGTWTCTAAT-3’) with Illumina adapter overhang sequences (underlined) (Ramadan et al. 2016, Tawfik et al. 2018). A total volume of 25 µl PCR mixture was prepared for each sample consisting of 12.5 µl of 2× KAPA HiFi Hotstart ReadyMix (KAPA Biosystems, Wilmington MA, USA), 5 µl each of 10 µM forward and reverse primers and 2.5 µl of template DNA. The PCR reaction was performed on Alpha Cycler 1 PCRmax with the following protocol: initial denaturation for 3 min at 95°C, 35 cycles of 30 sec at 95°C, 30 sec at 55°C and 30 sec at 72°C, with a final elongation at 72°C for 5 min. The PCR products were purified using 0.7x volume ratio of Kapa pure beads (KAPA Biosystems, USA). Then, the second PCR was conducted for indexing amplicons from the first PCR using Nextera XT Index Kit v.2 (Illumina Inc, USA): 5 µl of Index 1 and Index 2 primers were added to 1 µl of purified PCR product and 12.5 µl of 2× KAPA HiFi Hotstart ReadyMix. Optimised PCR conditions for 12 cycles are as follow: polymerase activation for 3 min at 72°C, initial denaturation for 30 sec at 95°C, 12 cycles of denaturation for 10 sec at 95°C, 30 sec at 55°C and 30 sec at 72°C, with a final elongation at 72°C for 5 min. The PCR products were examined by electrophoresis using 1.5% agarose gel in 1x TAE buffer. Next, sizes of PCR amplicons were analysed using LabChip GX Touch and a representative result from the E-gram with a clear peak at 652 bp is shown (See Suppl. material 1).

### Quantification and NGS

The purified amplicons containing the full-length Illumina adapters and unique barcodes were quantified using quantitative PCR (qPCR). This reaction was conducted using KAPA SYBR FAST qPCR Master Mix (KAPA Biosystems, USA). A total volume of 20 µl PCR mixture was prepared that contains 10 µl KAPA SYBR FAST qPCR Master Mix, 2 µl primer premix, 4 µl indexed-amplicon and 4 µl RNase-free distilled water. The PCR reaction was performed on a Eco48 Real Time PCR system (PCRmax) under the following conditions: 95°C for 5 min, followed by 25 cycles of 95°C for 40 sec, 60℃ for 2 min, 72°C for 1 min, with a final extension step at 72°C for 7 min. Normalisation is crucial for the success of sequencing, which equalises the concentration of DNA libraries for multiplexing in order to obtain similar number of reads from each sample. Based on the qPCR and Qubit quantification data, the amplicons were normalised and pooled into a single library for NGS. The multiplexed library consists of 300 µl pooled amplicons (1.3 pM) and 200 µl PhiX Control kit (1.3 pM). The sequencing length is 2 x 151 cycles using a MiniSeq High Throughput Reagent Kit on an Illumina Miniseq platform (Illumina Inc, USA). Sequencing was conducted at the Evolutionary and Conservation Genetics Laboratory of Department of Technology and Natural Resources, Universiti Tun Hussein Onn Malaysia.

### Sequencing data, bioinformatic and statistical analyses

All next-generation sequence data were deposited into National Center of Biotechnology Information (NCBI), under Sequence Read Archive (SRA) accession numbers; SRR16796807, SRR16798027, SRR14798122, SRR16805065, SRR16805071, SRR16805076, SRR16805547 and SRR16805567. Quality filtering and demultiplexing of generated sequences were performed using CLC Genomic Workbench software (CLC) (Qiagen, USA). An initial assessment of quality scores of the sequencing data was conducted on FASTQ files. Aligned sequences were then clustered into operational taxonomic unit (OTUs) defined by 97% similarity and OTUs were aligned using MUSCLE tool in CLC. Rarefaction curves were then plotted with the OTUs observed with a given sequencing depth using CLC. OTUs were given taxonomic assignment using SILVA v.138 database and these trees were used for the calculation of alpha diversity indices (Chao1, Shannon, Simpson and Evenness) in all samples from each island population as described in AST 3 software (Hammer et al. 2001). Beta diversity (weighted UniFrac distance) metrics were calculated to describe the dissimilarities by considering both the evolutionary distances and the frequency of occurrences of bacterial phylotypes observed amongst populations (Phillips et al. 2012). Principal coordinate analysis (PCoA) was illustrated, based on the UniFrac distances to portray the relationship amongst populations and a Venn diagram was generated to show the number of unique and shared OTUs amongst the populations at 97% similarity. Based on the Bray-Curtis measurements, heatmaps with a dendrogram were generated with the weighted pair clustering. The correlation of microbial diversity (genera) amongst the populations was then measured using the Pearson correlation coefficient with a significance threshold of p < 0.05 using PAST 3 software, supported by generation of cold-hot plot figure.

## Data resources

**Sample collection**

**Figure 2.**
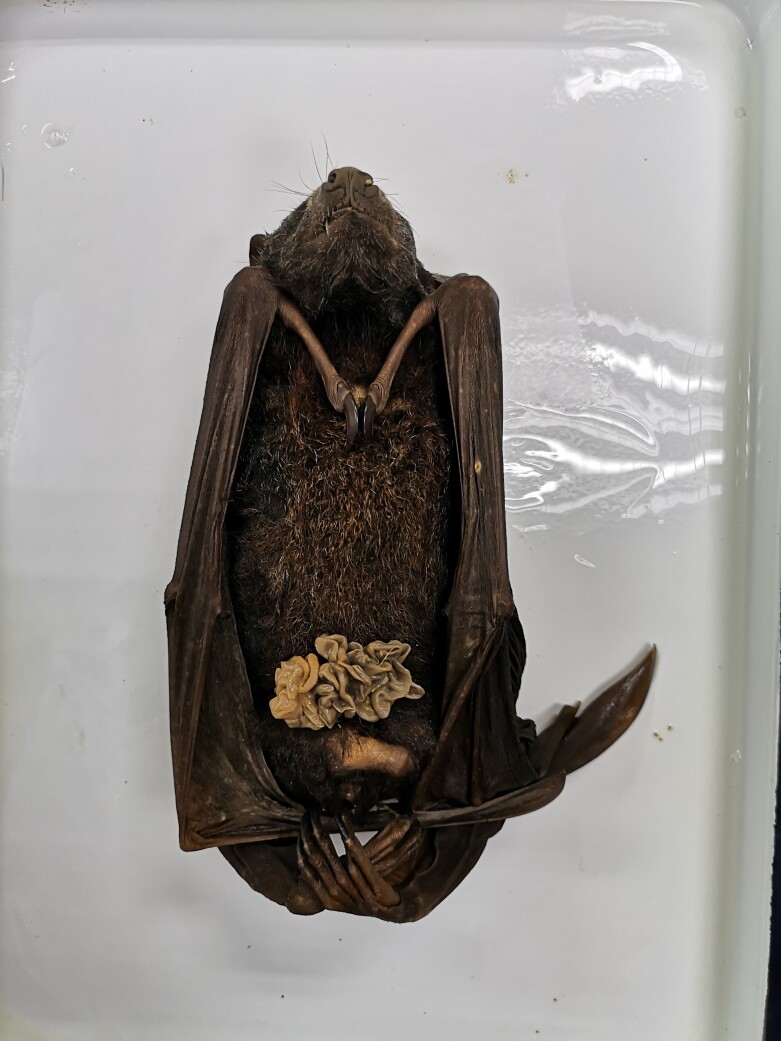
Dissection of Island Flying Fox.

A total of eight samples were collected and used in this metabarcoding analysis (Table 1). The Island Flying Foxes were captured and dissected with proper biosafety equipment in laboratories and a portion of the intestine content was collected (Daniel et al. 2013). An incision was made across the abdomen using a scalpel and surgical scissors. The intestinal content was then removed by gently squeezing with the swabs and sterile surgical forceps under aseptic conditions. Intestinal tissue (about 1 cm long) was used for total community DNA extraction (Fig. 2).

## Results

**Figure 3.**
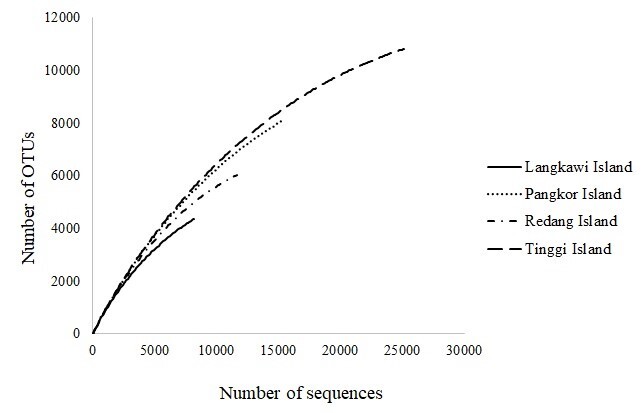
The rarefaction curve for OTUs defined at 97% similarity for different populations of *P.hypomelanus*. Vertical axis shows operational taxonomic units and the horizontal axis shows the number of samples sequenced.

A total of 80,472 sequences (<300 base pairs) in bacterial 16S rRNA gene OTUs were identified, ranging from 11,783 to 28,081 sequences from eight samples of each population (Table 2). Note: the final data were obtained after excluding low-quality sequence reads, clustering and the chimera removal. We also suggest future study to increase sequencing depth to ensure the total microbiome diversity of this species is successfully elucidated. At the 97% similarity cut-off, a total of 25,658 bacterial OTUs were produced, ranging from 811 to 10,849 OTUs for each sample. The alpha diversity index of OTUs, observed in island populations, ranges from 8.106 to 8.916 and 0.9995 to 0.9997 of richness estimated by Shannon H and Simpson 1-D, respectively (Table 2). Based on the Shannon and Simpson diversity indices, Redang Island showed the highest diversity of OTUs (H = 8.916, 1-D = 0.9997), followed by Langkawi Island (H = 8.694, 1-D = 0.9997), Pangkor Island (H = 8.438, 1-D = 0.9996) and Tinggi Island (H = 8.106, 1-D = 0.9995) (Table 2). Similar findings were observed in rarefaction analysis of OTUs from each population (Fig. 3). Three island populations revealed increasing rarefaction curves which indicate the bacterial richness analysis was complete without being biased by the number of sequences analysed. However, samples from Langkawi Island exhibited lower sequencing depth than other populations.

**Figure 4.**
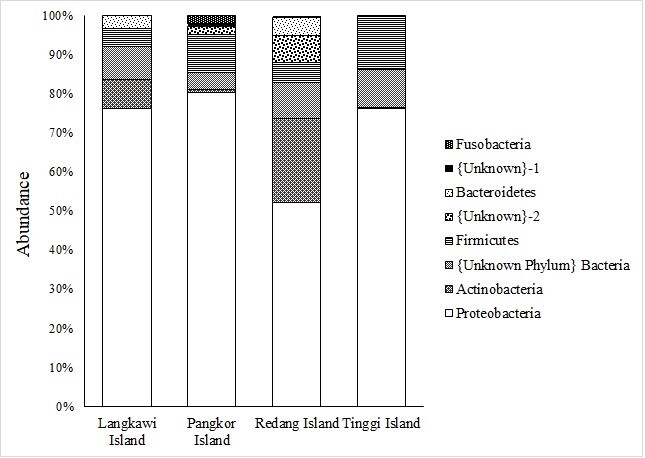
Phylum level distribution in the bacterial community between populations. Vertical axis shows locality and the horizontal axis shows the percentage of relative abundance.

**Figure 5.**
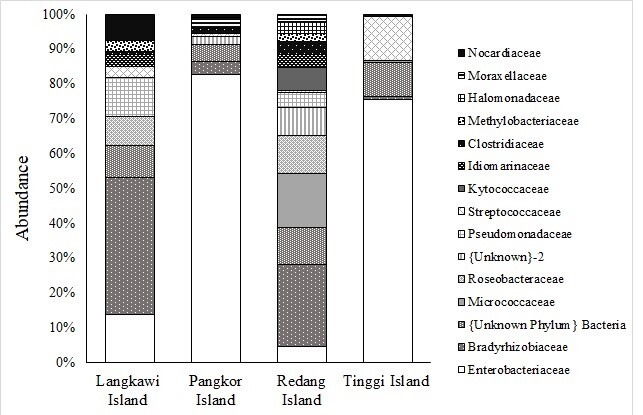
Family level distribution in the bacterial community between populations. Vertical axis shows locality and the horizontal axis shows the percentage of relative abundance.

**Figure 6.**
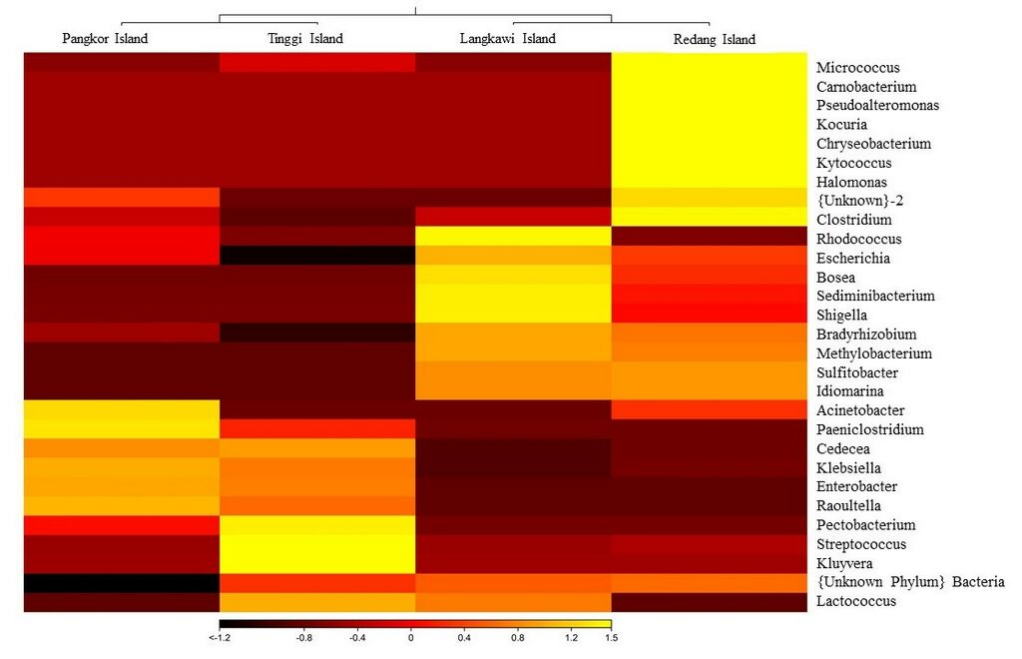
Heatmap with a dendrogram at the genus level using a gradient heatmap (over 1% of the microbiome). The 30 most predominant genera were used in hierarchical clustering to evaluate the relationships amongst four populations of *P.hypomelanus* using weighted pair clustering, based on Bray-Curtis measurements. The lighter colour indicates higher abundance.

The OTUs were successfully aligned to eight phyla, 44 families, 61 genera and 94 species. The relative abundance of various microbial taxa differed significantly amongst populations. The Proteobacteria was the dominant phylum and was found across the populations (Langkawi Island, 76.15%; Pangkor Island, 80.42%; Redang Island, 52.09%; and Tinggi Island, 76.27%) (Table 3). It was followed by other phyla, namely Actinobacteria and Firmicutes. Other phyla, including Bacteroidetes and Fusobacteria, were found in relatively low abundance, less than 5.0% (Fig. 4). Additionally, at the family level, Enterobacteriaceae and Bradyrhizobiaceae were the most predominant family found in overall sequences (Table 4). Other families including Micrococcaceae, Roseobacteraceae, Pseudomonadaceae, Streptococcaceae, Kytococcaceae, Idiomarinaceae, Clostridiaceae, Methylobacteriaceae, Halomonadaceae, Moraxellaceae and Nocardiaceae were found in relatively low proportions, less than 10.0% (Fig. 5).The 30 most predominant genera were analysed by hierarchical clustering to evaluate the relationship amongst four populations of *P.hypomelanus* and results are shown in the gradient heatmap with a dendrogram at the genus level (Fig. 6).

**Figure 7.**
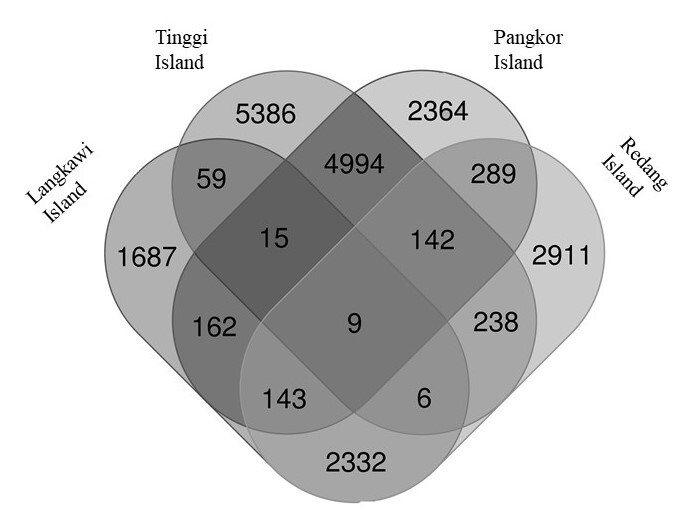
The Venn diagram illustrated the number of shared OTUs at the 97% similarity amongst four populations. Intersection part between circles indicated the number of shared OTUs.

**Figure 8.**
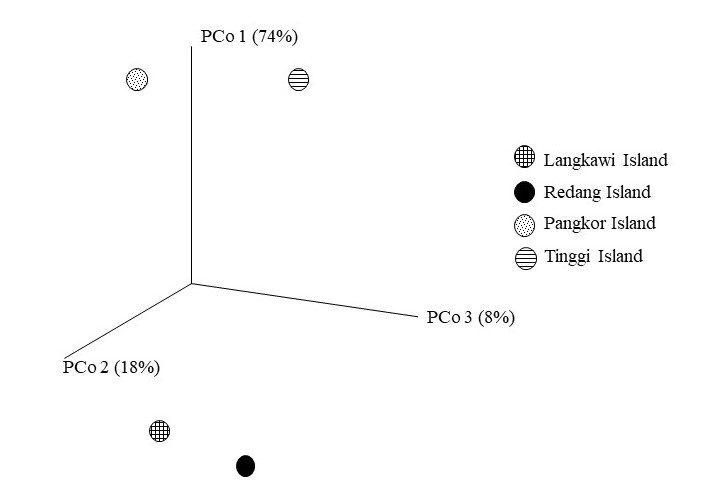
A three-dimensional plot of weighted UniFrac, based principal coordinate analysis (PCoA). The plot was created using the pairwise weighted UniFrac distances which account for microbial species richness and evenness where (PC1 is variability at 74%, PC2 is variability at 18% and PC3 is variability at 8%). Different shape indicates the different locality.

Based on the Venn diagram in Fig. 7, nine bacterial OTUs were shared amongst the four populations. Tinggi Island has 5386 OTUs with unique sequences, largest amongst all populations, followed by Redang Island (2911), Pangkor Island (2364) and Langkawi Island (1687). Similarly, based on UniFrac distances, Redang and Langkawi Islands clustered closely in PCoA analysis, indicating similar bacteria community patterns between two populations (Fig. 8).

### Correlation and Significance of Relationships between Populations

**Figure 9.**
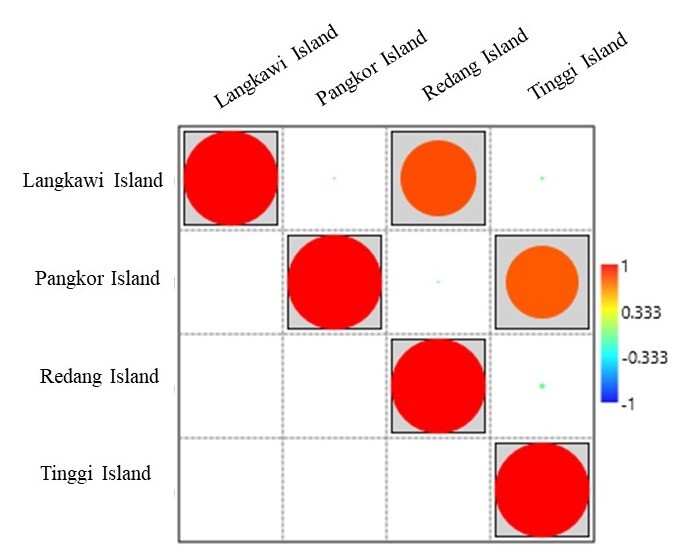
The cold-hot plot shows the correlation between the bacterial community (genera) amongst four island populations.

Principal coordinate analysis (PCoA), based on UniFrac, revealed that clustering of samples was according to the grouping of the 16S rRNA dendrogram (Fig. 8). Correlations between microbiomes at different populations were assessed using Pearson’s correlation analysis. The strongest correlations (r = 0.80, p < 0.05) were detected between the Redang Island and Langkawi Island in all samples, followed by Pangkor Island and Tinggi Island (r = 0.76, p < 0.05) (Table 5). Furthermore, by cold-hot plot, the strength of correlation between populations was scaled to a range from −1 to 1. Langkawi Island-Redang Island and Pangkor Island-Tinggi Island have strong correlations as their r value is close to 1 (Fig. 9).

## Discussion

### Diversity and abundance of gut microbiome of P.hypomelanus

The Island Flying Fox is of particular interest to researchers because bats are a group of volant mammals that have unique evolutionary adaptation to habitats. Recently, high-throughput sequencing technology has become available and provided an efficient tool for analysing the relationships between microorganisms that are thought to influence their species diversity and functions (Phillips et al. 2012). The purpose of the current study was to provide the first documentation on gut microbiome in *P.hypomelanus* from different island populations in Peninsular Malaysia.

In this study, we acquired data from bacteria in eight phyla, 44 families and 61 genera. This highlights the advantages of using high-throughput sequencing compared to culture-based approaches in studying bacterial communities (Wang et al. 2014, Wang et al. 2015). We observed that Proteobacteria phylum are predominant, confirming the stable nature of these phyla in bat gut environment from all populations. However, these same bacterial classes have also been observed in the intestinal microbiotas of other mammals, such as primates, buffalo and camel (Ochman et al. 2010, Bhatt et al. 2013, Patel et al. 2014). This suggests that these specific bacteria groups are especially well adapted to the environment in mammals’ intestine.

Moreover, two families of gut bacteria (Enterobacteriaceae and Bradyrhizobiaceae) were most abundant in the group of Proteobacteria. These bacterial families are facultative anaerobes and function in glucose fermentation (Banskar et al. 2016a). In contrast, Streptococcaceae show a small percentage of Firmicutes group (~13%), being the second most dominant family in Tinggi Island. Bacterial phyla, including Firmicutes, have been associated with digestion of diets rich in fibre and fermentable carbohydrates (Carrillo-Araujo et al. 2015, Chen et al. 2018). According to Carrillo-Araujo et al. (2015), the effects of bacterial phyla Firmicutes on the ecology and physiology of wildlife are not well-understood. Thus, it would be very important to understand the implications of these bacteria in metabolism of their hosts with high carbohydrate intake.

The general composition of gut microbiota in bats is strikingly different from that of other mammalian gut microbiomes, which are generally dominated by Firmicutes. Interestingly, the relatively high abundance of Proteobacteria in the chiropteran gut is similar to that found in the avian gut (Hird et al. 2015). Regardless of diet, the distal gut of bats is dominated by bacteria in the family Enterobacteriaceae (phylum Proteobacteria), although fruit bats hosted a larger amount of Clostridiaceae (phylum Firmicutes) and Streptococcaceae (phylum Firmicutes) than insectivorous bats, a finding previously observed in Neotropical bats (Phillips et al. 2012). Many bacterial species in the Lactobacillales are found on fermenting fruits (Splittstoesser 1982, Endo et al. 2018); thus, the presence of Streptococcaceae (order Lactobacillales) in the fruit bat gut may be due to ingestion rather than established residence, a possibility that requires further investigation.

From 61 genera isolated from the intestinal content of Flying Fox, some of the genera contain non-pathogenic species and a common presence of potentially pathogenic microbes, including *Pseudomonas*, *Escherichia*, *Streptococcus* and *Clostridium*. Previous studies have reported a similar bacteria genus associated with bats, although some genera were not isolated from this study (Anand and Sripathi 2004, Mühldorfer et al. 2010, Di Bella et al. 2014). This could be due to the limited number of bacterial isolates, the small sample size used or the phylogenetics of the host itself (Warren and Witter 2002). Thus, a higher resolution method needs to be used to establish strain identity. Meanwhile, the genera *Enterobacter*, *Pseudomonas* and *Klebsiella*, isolated in this study, were previously found in the rectum of other fruit bats, such as *Cynopterusbracyhotis* (Daniel et al. 2013). Most gut microbiota in mammals play a role in breaking down carbohydrates into short fatty acid chains (acetate, propionate and butyrate) which provide energy and nutrients for the organisms (Caroline et al. 2003, Macfarlane and Macfarlane 2003, Samuel et al. 2008). For example, *Enterobacter* breaks down most sugars including xylose, which is one of the most abundant components in plants (Rastall 2004). Besides, *Klebsiella* are also known to exhibit cellulolytic properties (Anand and Sripathi 2004).

### Comparison of diversity and abundance of gut microbiome of P.hypomelanus between islands in Peninsular Malaysia

The results of our metabarcoding analysis showed both variation in diversity and species composition of bacterial communities in *P.hypomelanus* amongst different island populations. Overall, the population from Redang Island has a relatively high diversity index (H') of gut microbiome compared to other populations. Meanwhile, the population from Tinggi Island has the lowest diversity index (H') of gut microbiome. Low bacterial diversity is associated with poor physical fitness, while high diversity indicates good health (Scott et al. 2015). In addition, populations from Pangkor Island and Tinggi Island have higher abundance of Proteobacteria than the other populations, at 80.42% and 76.27%, respectively.

Langkawi Island, located in Kedah, has a land area of 328 km^2^, while Redang Island in Terengganu is 42 km^2^, Pangkor Island in Perak is 18 km^2^ and Tinggi Island in Johor is 15 km^2^. In this study, we tested the hypothesis that the Flying Fox, occupying islands with different habitat quality and environment, may develop different gut microbiome. Langkawi and Redang Islands have a larger island size compared to Pangkor Island and Tinggi Island. Animals occupying large areas of habitats consumed more diverse diets and consequently sustain high gut microbiome richness and diversity (Amato et al. 2013). Therefore, we predicted large islands tend to have lower extinction rate because they usually have more food resources and more diverse habitats for residential species.

Residential populations around the islands indirectly affect the abundance and diversity of gut microbiome of *P.hypomelanus*. Sampling areas in Langkawi Island and Redang Island were better protected from interference by humans compared to those in Pangkor Island and Tinggi Island. Besides, Tinggi Island comprises three villages, namely Kampung Tanjung Balang, Kampung Pasir Panjang and Kampung Sebirah Besar, with a large number of residents. Habitats with high population density and scarce food sources due to human disturbance may affect the diet of *P.hypomelanus* on the Island. As a result, their diets deviate from the typical dietary habits, which can be associated with shifts in microbiome composition. In general, animals in disturbed habitats consume different types of food from animals in less disturbed areas (Flaherty et al. 2010, Abbas et al. 2011, Chaves et al. 2012). Moreover, inhabiting disturbed habitats may lead to dysbiosis, such as lower microbial richness and diversity that may affect host nutrition and health (Stumpf et al. 2016). Overall, gut microbiome can affect hosts by altering fat storage during food scarcity and environmental changes (Faith et al. 2011, David et al. 2014). Results from this study suggest that the diet of flying fox in minimally disturbed habitats in Redang Island appears to promote the acquisition and maintenance of a diverse gut microbiome.

The gut microbiomes in the populations of Langkawi Island and Redang Island were found to be closely related, supported by the shared number of OTUs displayed by the Venn diagram. Besides, one of the PCoA axes correlate which shows island populations (Redang and Langkawi) clustering together and supported with low percentage of variability, as there were no significant differences in bacterial diversity of the gut microbiota. As suggested in previous studies on flying vertebrates (bats and birds), convergent adaptations driven by flight may influence digestive physiology, such as increasing paracellular absorption and accelerating the transit time of food through the gut (Caviedes-Vidal et al. 2008, Canals et al. 2011, Price et al. 2015). These physiological adaptations to flight may, in turn, affect the nature and composition of microbial communities in flying vertebrates. Moreover, flying is an energetically demanding form of locomotion that requires the animal to have a small size of gut. Compared to other non-flying mammals, bats have relatively smaller gastrointestinal (GI) tracts and reduced intestinal tissue which can help to minimise flight mass (Caviedes-Vidal et al. 2007, Caviedes-Vidal et al. 2008, Strobel et al. 2015). A recent study by Lutz et al. (2019) also reported that the energetic demands of flight place significant constraints on gut physiology, which, in turn, restricts the microbial community in the gut.

Taken together, these factors have shaped the structure of the community, which revealed bacterial communities between island populations at larger spatial scales in Peninsular Malaysia. Our results revealed important mechanisms that are critical for improving our knowledge of host-microbe interactions in Island Flying Fox populations. This information will serve as a basis for conservation efforts and better assessment of the impact of human activity on animal health and zoonotic disease managements. However, future research is needed to identify specific plant taxa in the diet of *P.hypomelanus* at finer geographic scales to help promote the healthy gut microbiome in *P.hypomelanus* in Peninsular Malaysia.

## Conclusions

In conclusion, we have outlined the diversity and distribution of bacteria isolated from the gut of Island Flying Fox across four populations in Peninsular Malaysia. Given that collection localities were separated by geographical distance and differ in island size, an unexpected observation from this study was that environment of island populations apparently influences the gut microbiome, suggesting the microbiota observed in bats is not driven by host evolution, but rather by ecological features.

## Supplementary Material

62A4B54D-5495-5B19-BC37-ADF14D8AB1CE 10.3897/BDJ.10.e69631.suppl1 Supplementary material 1 E-gram Data type Image File: oo_566676.jpeg https://binary.pensoft.net/file/566676 Nur Syafika Mohd-Yusof, Muhammad Abu Bakar Abdul-Latiff, Abd Rahman Mohd-Ridwan, Aqilah Sakinah Badrulisham, Nursyuhada Othman, Salmah Yaakop, Shukor Md-Nor and Badrul Munir Md-Zain

## Figures and Tables

**Table 1.** List of samples and localities used for microbiome analysis.

#	Sample code	Locality	Source of isolation
1	DA	Dangli Island, Langkawi	Intestine swabs
2	DB	Dangli Island, Langkawi	Intestine swabs
3	PB	Pangkor Island, Perak	Intestine swabs
4	PC	Pangkor Island, Perak	Intestine swabs
5	RA	Redang Island, Terengganu	Intestine swabs
6	RB	Redang Island, Terengganu	Intestine swabs
7	TA	Tinggi Island, Johor	Intestine swabs
8	TB	Tinggi Island, Johor	Intestine swabs

**Table 2.** Numbers of effective 16S rRNA gene sequences, numbers of observed OTUs, alpha diversity indices (Chao1, Shannon and Simpson) and Evenness for the bacterial community in four island populations.

Population	Sequences	OTUs*	Chao1	Evenness (e)	Shannon (H)		Simpson (1-D)
Langkawi Island	28081	4413	1.14E+04	0.735		8.694	0.9997
Pangkor Island	15362	811	7372	0.761		8.438	0.9996
Redang Island	11783	6070	1.23E+04	0.687		8.916	0.9997
Tinggi Island	25246	10849	6234	0.751		8.106	0.9995
Overall	**80472**	**25658**					

**Table 3.** Relative abundance of microbiome communities at the phylum level in different populations.

#	Phylum	Langkawi Island (%)	Pangkor Island (%)	Redang Island (%)	Tinggi Island (%)
1	Proteobacteria	76.15	80.42	52.09	76.27
2	Actinobacteria	7.53	0.70	21.62	0.32
3	{Unknown Phylum} Bacteria	8.37	4.20	9.09	9.49
4	Firmicutes	4.60	9.79	5.28	13.92
5	{Unknown}-2	0.00	2.10	6.88	0.00
6	Bacteroidetes	3.35	0.00	4.42	0.00
7	{Unknown}-1	0.00	0.70	0.61	0.00
8	Fusobacteria	0.00	2.10	0.00	0.00

**Table 4.** Relative abundance of microbiome communities at the family level in different populations.

#	Family	Langkawi Island (%)	Pangkor Island (%)	Redang Island (%)	Tinggi Island (%)
1	Enterobacteriaceae	13.89	82.68	4.65	75.75
2	Bradyrhizobiaceae	39.35	3.94	23.70	0.66
3	{Unknown Phylum} Bacteria	9.26	4.72	10.44	9.97
4	Micrococcaceae	0.00	0.00	15.51	0.33
5	Roseobacteraceae	0.83	0.00	11.14	0.00
6	{Unknown}-2	0.00	2.36	7.90	0.00
7	Pseudomonadaceae	11.11	0.00	4.23	0.00
8	Streptococcaceae	3.24	0.79	0.56	12.62
9	Kytococcaceae	0.00	0.00	6.63	0.00
10	Idiomarinaceae	3.24	0.00	3.81	0.00
11	Clostridiaceae	0.93	1.57	3.67	0.66
12	Methylobacteriaceae	3.24	0.00	2.40	0.00
13	Halomonadaceae	0.00	0.00	3.39	0.00
14	Moraxellaceae	0.00	3.15	1.97	0.00
15	Nocardiaceae	7.41	0.79	0.00	0.00

**Table 5.** The correlation coefficient values (Pearson r) and p-values (**bold**) of bacterial community (genera) amongst island populations.

	Langkawi Island	Pangkor Island	Redang Island	Tinggi Island
Langkawi Island		**0.89703**	**1.54E-14**	**0.83656**
Pangkor Island	0.016919		**0.90198**	**8.77E-13**
Redang Island	0.79706	-0.016102		**0.71778**
Tinggi Island	-0.026967	0.76307	-0.047228	
