# Supplementary figures and images for: First report on metabarcoding analysis of gut microbiome in Island Flying Fox (Pteropushypomelanus) in island populations of Malaysia
Source: Biodivers Data J. 2022 Mar 22;10:e69631. doi: 10.3897/BDJ.10.e69631 (PMC9848629; doi:10.3897/BDJ.10.e69631)

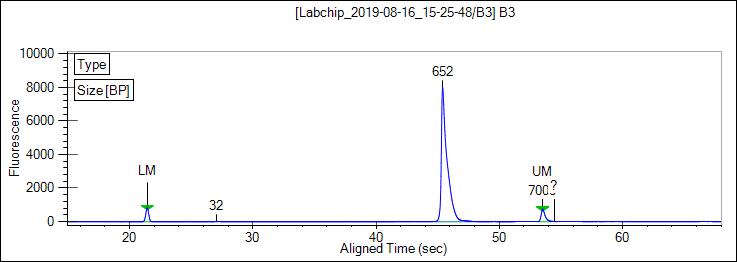

Supplement: Supplementary material 1 — E-gram [file bdj-10-e69631-s001.jpeg]
